# Direct observation of the Si(110)-(16×2) surface reconstruction by atomic force microscopy

**DOI:** 10.3762/bjnano.11.157

**Published:** 2020-11-19

**Authors:** Tatsuya Yamamoto, Ryo Izumi, Kazushi Miki, Takahiro Yamasaki, Yasuhiro Sugawara, Yan Jun Li

**Affiliations:** 1 Department of Applied Physics, Graduate School of Engineering, Osaka University, 2-1 Yamadaoka, Suita, Osaka 565-0871, Japan; 2 Department of Electrical Materials and Engineering, University of Hyogo, Shoya 2167, Himeji, Hyogo 671-2280, Japan; 3 Institute for Nanoscience Design, Osaka University, 1-2 Machikaneyama, Toyonaka, Osaka 560-0043, Japan

**Keywords:** atomic force microscopy (AFM), noncontact atomic force microscopy (NC-AFM), Si(110), Si(110)-(16×2)

## Abstract

The atomic arrangement of the Si(110)-(16×2) reconstruction was directly observed using noncontact atomic force microscopy (NC-AFM) at 78 K. The pentagonal structure, which is the most important building block of the reconstruction, was concluded to consist of five atoms, while only four or five spots (depending on tip bias) have been reported with scanning tunneling microscopy (STM). Single atoms were determined to exist near step edges between upper and lower terraces, which have not been reported using STM. These findings are key evidence for establishing an atomic model of the Si(110)-(16×2) reconstruction, which indeed has a complex structure.

## Introduction

The Si(110) surface, which is one of the low-index Si planes, has been attracting growing interest in the fields of industrial technology and surface science. From an industrial application perspective, it has been considered to be a promising material for p-type high-performance metal–oxide–semiconductor field-effect transistors (p-MOSFETs) [1–2] because the hole mobility of Si(110) is twice that of the other Si planes [3]. For surface science research, Si(110) has been used as a template substrate for self-assembled nanowires [4–6], nanomeshes [7], and nanodots [8–9]. Particularly, the Si(110)-(16×2) reconstructed surface is considered to be an ideal 1D template [4,7,9]. Since this reconstructed surface is reported to be two dimensionally chiral, it has been the subject of many investigations, for example, in efforts to control the chirality for reliable production of nanowires and other nanostructures [7,10–13].

By annealing below 700 °C [14], the Si(110)-(16×2) reconstruction is formed over large areas on the Si(110) surface. It has been widely investigated by reflection high-energy electron diffraction (RHEED) analysis [14–15], scanning tunneling microscopy (STM) [15–24], scanning transmission electron microscopy (STEM) [25], and photoelectron spectroscopy (PES) [22,26–27]. The 16×2 reconstruction has a striped structure with upper and lower terraces and with boundaries of monatomic steps. In STM images a pair of pentagons is revealed on upper and lower terraces in a unit cell. The pentagon has five spots at a negative tip bias and it has four spots at a positive tip bias in STM observations. Monatomic steps (both up and down steps) and the pentagon are two characteristic features of Si(110)-(16×2), but the details of these atomic structures are still unknown. Because most of the knowledge of the reconstruction has been deduced from STM observations, information regarding the atomic structure is lacking. Similar step [28–29] and pentagonal structures [30–32] have been found on other group IV surfaces.

Although considerable research has been carried out to determine the atomic structure of the Si(110)-(16×2) reconstruction, no good structural model has been established. Specifically, the interpretation of pentagons remains controversial. As the structural model, the adatom-tetramer-interstitial (ATI) model [21,33–34] had been widely accepted for many years. However, recently it was found that the experimental results obtained by STM [22–23] and PES [22,27] contradict the electronic state of the ATI model [35]; thus it has become doubtful whether a tetramer-interstitial pentagon can be used as a building block component. As an alternative to the ATI model, the adatom-buckling (AB) model [22] and the tetramer heptagonal- and tetragonal-ring (THTR) structure model [36] were proposed, but their atomic structures have not been confirmed completely because information regarding the atomic structure of the reconstruction that includes both pentagons and step edges is insufficient.

In this study, the Si(110)-(16×2) reconstruction will be investigated by atomic force microscopy (AFM) at 78 K to directly observe the atomic arrangement of the reconstruction. In this work, we succeeded in obtaining high-resolution images of pentagons and step edges by AFM. In this work, we present key evidence to confirm the reconstruction model in which each pentagon consists of five atoms and single atoms exist around step edges, which has not been reported using STM.

## Methods

The experiments were performed using noncontact atomic force microscopy (NC-AFM) under ultrahigh vacuum (UHV) conditions, where the frequency modulation AFM (FM-AFM) method was used. The pressure was maintained below 3 × 10^−11^ Torr and the temperature was held at 78 K. As a probe, a commercially available Si cantilever was used, which was cleaned by Ar^+^ sputtering to remove the oxide and contamination on the tip. The deflection of the cantilever was measured by the optical beam deflection method. The topography of the surface was imaged while feedback electronics were used to adjust the tip–sample distance to keep the frequency shift constant. When the imaging became unstable, a bias voltage was applied between the tip and sample to eliminate the electrostatic force between the tip and sample. As a sample, p-doped Si(110) with a resistivity of 1–5 Ω·cm was used, which was cleaned by cycles of flushing at 1200 °C for 3 s and annealing at 650 °C for 30 min. The sample was heated by flowing an electric current in the 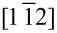 direction; thereby, a single-domain surface can be easily formed on the Si(110) surface [10].

## Results and Discussion

**Figure 1:**
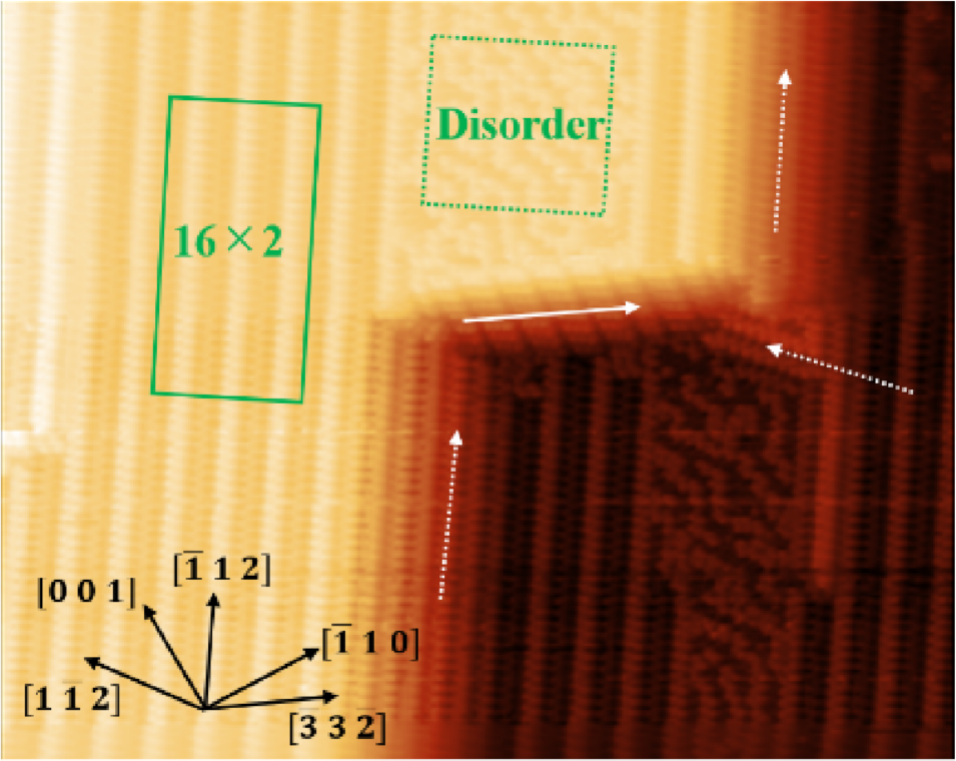
AFM image (100 × 80 nm^2^) of the 16×2 reconstruction on Si(110). Solid and dotted rectangles represent the 16×2 reconstruction and disorder regions, respectively. The solid arrow indicates the step in 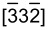 direction. The dashed arrows indicate the (17 15 1) and (15 17 1) facets in the 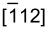 and 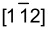 directions, respectively (*f*_0_ = 162.5 kHz, *A* = 5 nm, *V*_s_ = 0 mV, *T* = 78 K, Δ*f* = 18 Hz).

Figure 1 shows a typical AFM image of a Si(110) surface. The characteristic structures were observed in flat and step areas. In the flat area, the 16×2 reconstruction area (solid square) and the disorder area (dotted square) were mixed. The 16×2 reconstruction is known to have chirality with zigzag chains extending in the direction of 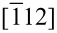 or 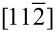 [13,21]. In this AFM image, zig-zag chains extending in the 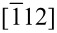 direction were dominant. However, during the process of sample cleaning, not only zig-zag chains extending in the 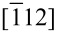 direction but also those extending in the 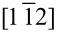 direction were also frequently dominant. Thus, the direction of the 16×2 reconstruction (or the direction in which zig-zag chains extend) was not related to the direction of current during sample heating, which is consistent with previous studies [12,22]. In the step area, two types of typical facets were observed: (17 15 1) and (15 17 1) [12,16] extending in the directions of 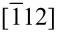 and 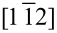, respectively, which are indicated by dashed arrows in Figure 1. The other facet extended in the direction of 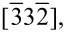 indicated by a solid arrow. This step showed stacked parallelogram-shaped units and was as common as the (15 17 1) vicinal step on this Si(110) surface and may be formed by a miscut [11].

**Figure 2:**
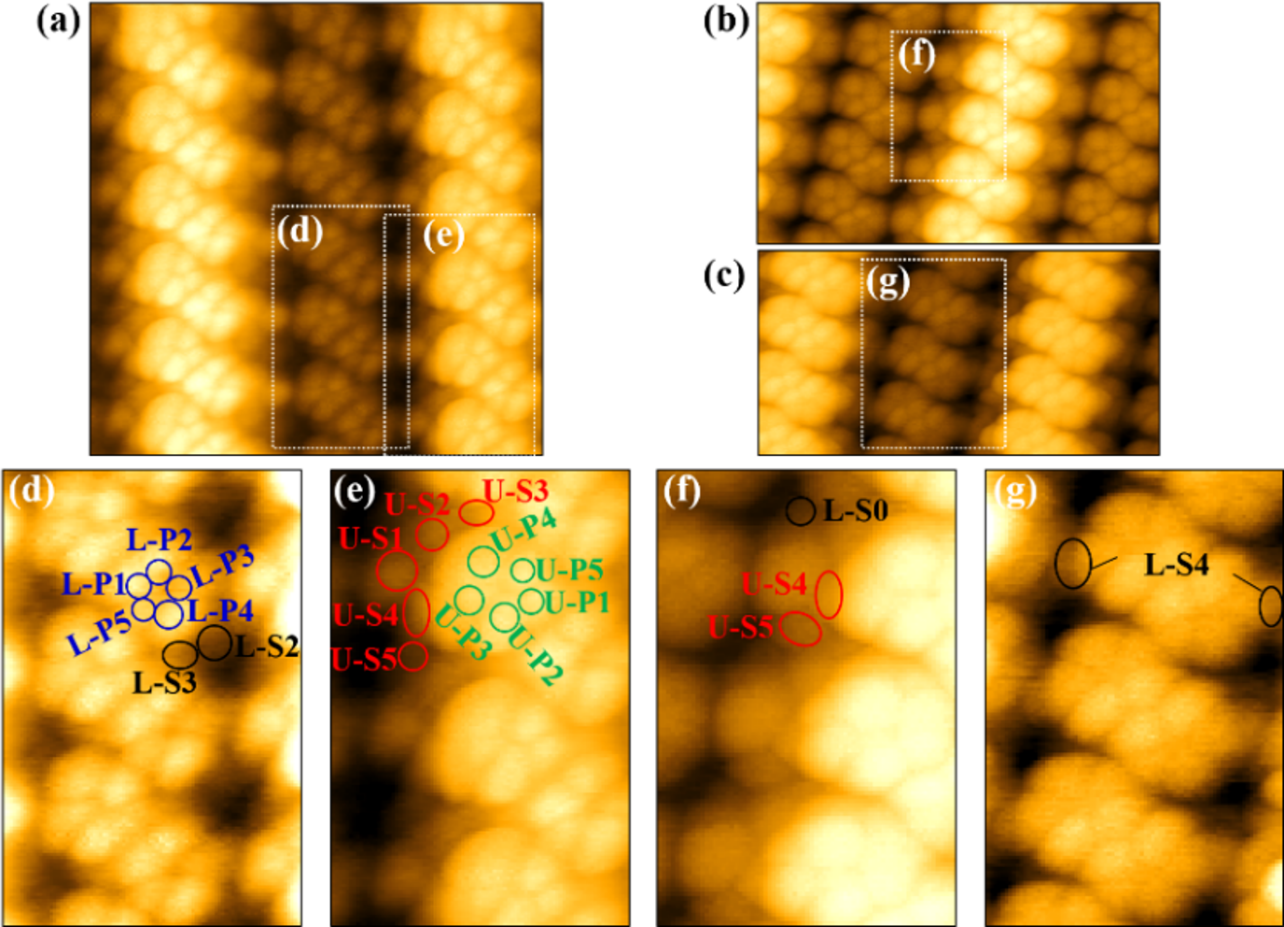
(a) Atomic-resolution AFM image (8 × 8 nm^2^) of the 16×2 reconstruction area shown in Figure 1 (*f*_0_ = 162.5 kHz, *A* = 5 nm, *V*_s_ = 0 mV, *T* = 78 K, Δ*f* = 18 Hz). (b), (c) AFM images of the 16×2 reconstruction, with a noticeably different appearance compared to (a) at the step edge (*f*_0_ = 162.0 kHz, *A* = 5 nm, *V*_s_ = 200 mV, *T* = 78 K, Δ*f* = 17 Hz). (d), (e), (f), (g) Enlarged images of (a), (b), and (c), indicated by white squares. The observed bright spots are marked with colored circles. The bright spots in the pentagonal structure are labeled as P1 to P5, and those at the step edge are labeled as S0 to S5. U- and L- denote those on the upper and lower terraces, respectively.

Figure 2a shows an AFM image of the 16×2 reconstruction area shown in Figure 1, and Figure 2d,e show the enlarged images of Figure 2a. Pairs of pentagons aligned in a zig-zag row were observed on the upper and lower terraces, which were also observed by STM [21]. Furthermore, several bright spots were observed at step edges. At the step edge, the structure is intricate and the observable bright spots were different depending on the tip state. Figure 2b and Figure 2c show AFM images of the 16×2 reconstruction that appears to be different from Figure 2a at the step edge. Figure 2f,g are enlarged images of Figure 2b,c, respectively. In all images, five bright spots aligned in a pentagonal structure, marked as U-P1, U-P2, U-P3, U-P4, and U-P5 on the upper terrace and as L-P1, L-P2, L-P3, L-P4, and L-P5 on the lower terrace, were observed. Because the atomic contrast on a Si surface comes from dangling bonds of Si atoms in AFM images [37], these five bright spots can be explained by the dangling bonds of five atoms. Therefore, it was concluded that the five bright spots in pentagonal formation observed for a negative tip bias in STM images correspond to five atoms, that is, they were not due to crosstalk between the topography and the electronic state in STM. With respect to the step edge, in Figure 2a,d,e, U-S1, U-S2, U-S3, U-S4, and U-S5 on the upper terrace, and L-S2 and L-S3 on the lower terrace were observed. In Figure 2b,f U-S4 and U-S5 were observed more clearly as compared to Figure 2a, and it was found that the height of U-S5 was lower than that of U-S4. In addition, L-S0 was observed on the lower terrace. In Figure 2c,g, L-S4, which is located behind L-P3 and U-S1, was observed on the lower terrace.

**Figure 3:**
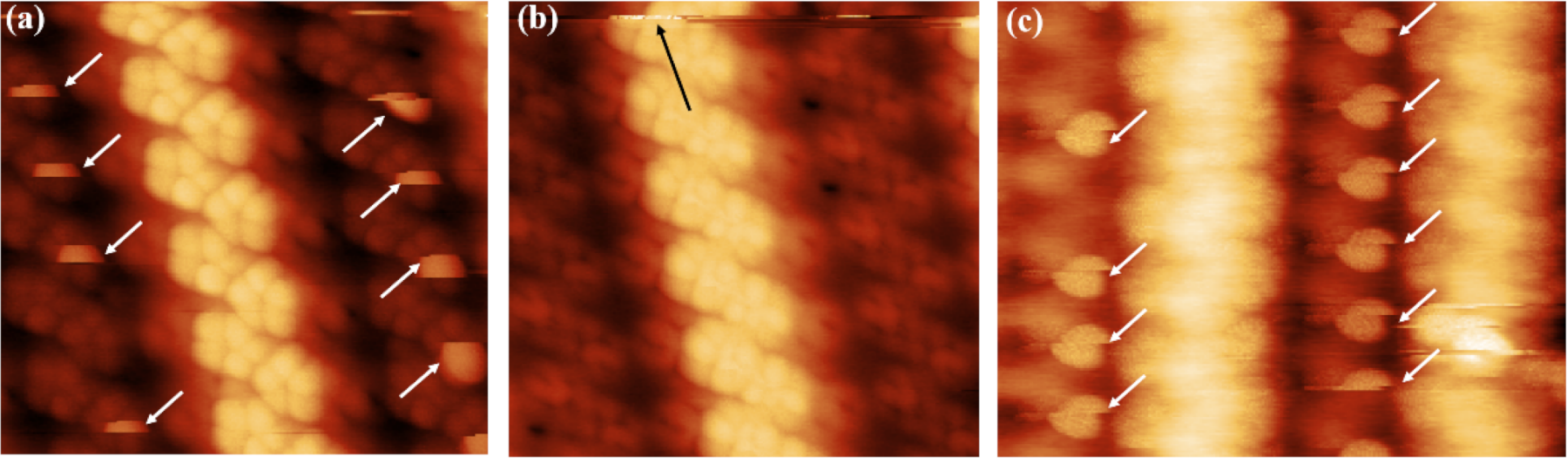
(a) An AFM image (7 × 6 nm^2^) of the 16×2 reconstruction surface with protrusions on L-P3 sites. White arrows indicate the protrusions. The fast scan direction is left to right, the slow scan direction is top to bottom. (b) An AFM image in the same area as (a). The tip state changed in the position indicated by the black arrow. The scan direction is the same as (a) (*f*_0_ = 162.0 kHz, *A* = 5 nm, *V*_s_ = 0 mV, *T* = 78 K, Δ*f* = 17 Hz). (c) An AFM image (10 × 8 nm^2^) of the 16×2 reconstruction surface with protrusions by using a different tip from Figure 3a (*f*_0_ = 155.9 kHz, *A* = 5 nm, *V*_s_ = 0 mV, *T* = 78 K, Δ*f* = 7 Hz).

When scanning, it was sometimes difficult to image a clean 16×2 reconstruction with atomic resolution. Figure 3a shows an AFM image of a 16×2 reconstruction with sudden protrusions on L-P3 sites (defined in Figure 2a). The direction of fast scan was left to right, and that of slow scan was top to bottom. It can be ruled out that there were adsorbates on L-P3 sites because the pressure was kept below 3 × 10^−11^ Torr and no evaporation occurred. Figure 3b shows an AFM image scanned just after Figure 3a, where the scan direction was the same as in Figure 3a. The state of the tip apex changed in the position indicated by a black arrow. The image appearance changed from Figure 3a and the sudden protrusions on L-P3 sites could no longer be observed. Two consecutive scans of Figure 3a and 3b indicated that these sudden protrusions were not adsorbates on L-P3 sites because they are observed in Figure 3a and not observed in Figure 3b. They can be explained by the fact that the L-P3 atom is pulled by the Si tip. Specifically, it can be considered that when the tip started to pass above L-P3, the tip pulled up only L-P3 atoms, which appeared as the protrusions on L-P3 sites in the AFM image, and that LP-3 atoms returned to their original positions after the tip finished passing by the L-P3 atoms.

Also, because sudden protrusions did not appear after the state of the tip apex changed, it can be concluded that the LP-3 atom state (that is, whether it is pulled up by the tip or not) depends on the state of the tip apex. Figure 3c shows the same phenomenon as Figure 3a when using a different tip and a different Si(110) sample. Given that this kind of sudden protrusion could be imaged even with different Si tips and different samples, we can conclude that this phenomenon originates from the property of this reconstruction, and is not a coincidence. This indicates that among the atoms in this reconstruction surface, only the L-P3 atom can be easily deformed by external force.

Next, we will identify atom positions, observed around step edges in the AFM image. We assign the Si lattice to the AFM image, referring to the model proposed by An et al. [21] and Setvín et al. [23–24]. First, we determined the 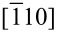 direction by assuming that the bright spot rows of U-S1, U-S2, and U-S3 are the zig-zag rows of the first layer Si atoms which buckle. Then, we assigned the 1st and 2nd layer Si rows with respect to this direction. Finally, we determined the Si lattice position so that the position of the first layer Si rows relative to the pentagons on the upper terrace and that of the second layer Si rows to the pentagons on the lower position are the same.

**Figure 4:**
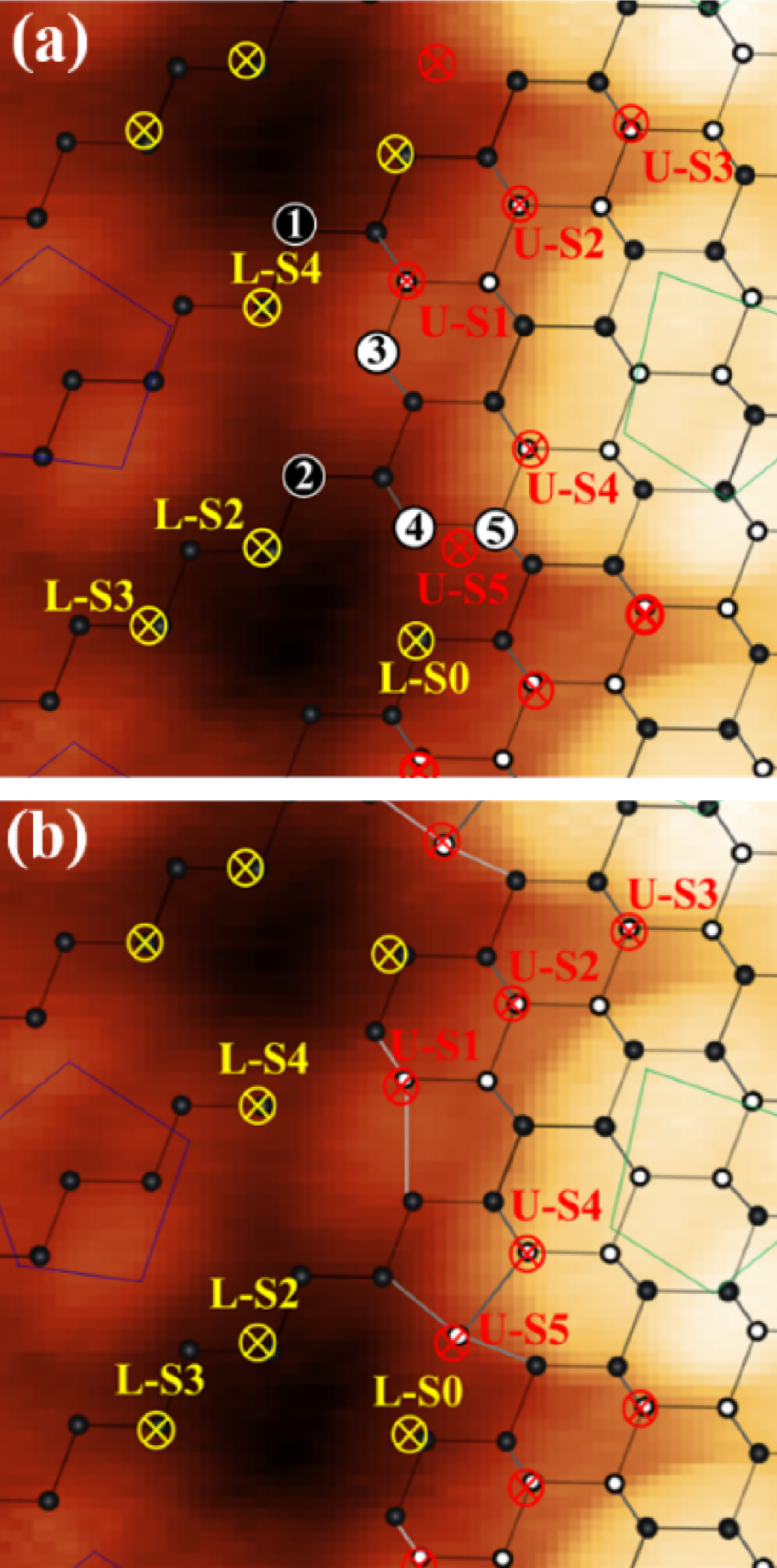
(a) An AFM image of the 16×2 reconstruction with the atomic lattice of the unreconstructed surface containing atomic steps. The rows of Si atoms in the first and second layers are indicated by white and black circles, respectively. Red and yellow crossmarks indicate the dangling bonds observed in AFM images on the upper terrace and lower terrace, respectively. Some atoms are labelled as 1 to 5 for clarification. (b) The atomic model with atoms 1, 3, and 4 removed from (a).

Figure 4a shows an atomic model of an unreconstructed Si(110) surface containing atomic steps. White and black circles indicate the first and second layer Si rows. In AFM images, the atomic contrast on a Si surface is due to the dangling bonds of the Si surface [37]; thus, we attribute the bright spots observed in AFM images to the dangling bonds of surface Si atoms. The bright spots, U-S1, U-S2, U-S3, U-S4, L-S0, L-S2, and L-S3, can be explained by the dangling bonds of atoms marked as crossmarks. In the following, we will distinguish between the bright spots and the atoms which generate these bright spots in AFM images with terminology such as “U-S1” and “U-S1 atom”, respectively. With respect to U-S5, the dangling bonds which generated the bright spot U-S5 should belong to atom 4 or 5. However, if atom 4 existed it would bond to the L-S0 atom, which would result in the saturation of the dangling bond of the atom [23]. Therefore, atom 4 must be removed from consideration and the bright spot of U-S5 should be attributed to the dangling bond of atom 5. When atom 4 is removed, atom 5 is located at the edge of the first layer Si row. To make atom 5 three-coordinated, it should bond with surrounding atoms as proposed by the atomic model [34] (shown in Figure 4b). In this case, the dangling bond of the U-S5 atom points obliquely to the surface, which can explain why the height of U-S5 was lower than that of U-S4 in the AFM image. Atom 3 is also the atom located at the edge of the first layer Si row. To make atom 3 three-coordinated, it should bond to the L-S4 atom, which would result in the saturation of the dangling bond of L-S4. To avoid saturating the dangling bond of L-S4, atom 3 must be removed. In this case, the U-S1 atom is located at the edge of the first layer Si row. To make U-S1 atom three-coordinated, it should bond with surrounding atoms as proposed by the atomic model [23] (shown in Figure 4b).

**Figure 5:**
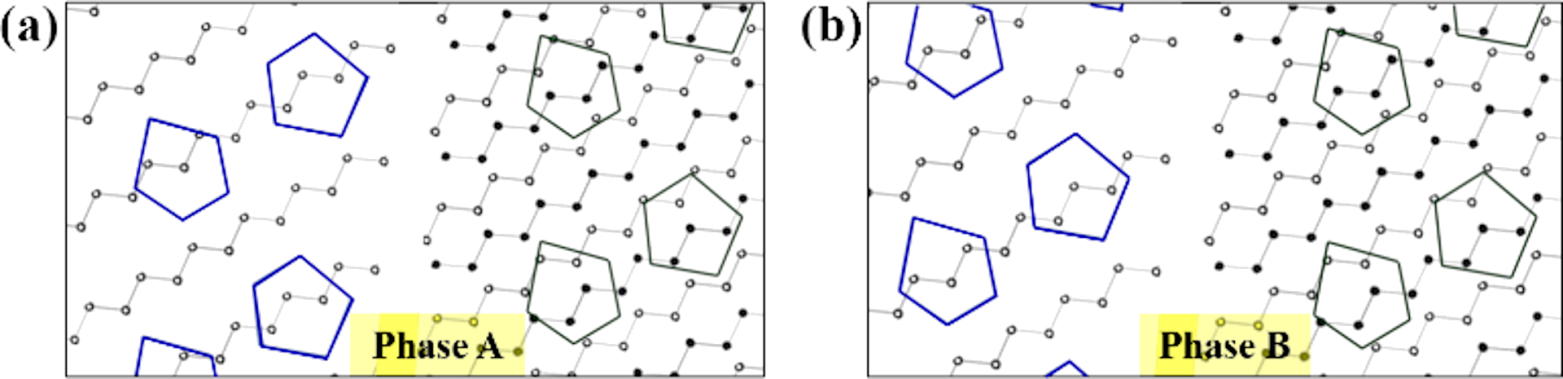
Models of phase relationships between pentagons on the upper terrace (green) and those on the lower terrace (blue) with atom 1 and 2 (defined in Figure 4) being removed. The first layer and second layer Si rows are indicated by black and white circles, respectively. (a) Phase A shows an actual phase relationship of the 16×2 reconstruction. (b) In phase B, pentagon pairs on the lower terrace are shifted by a half cycle (16×1) along the zig-zag row.

After atoms 3 and 4 are removed and the U-S5 atom and U-S1 atom are made three-coordinated, atom 1 and 2 are still three-coordinated and have dangling bonds extending in the direction of vacuum, but we could not observe any bright spots at these locations in the AFM images. In this case, there are two possible explanations: the atom does not exist at the location, or the atom simply could not be observed because the tip shape was insufficient. In the case of atom 1, removal of atom 1 can explain why only the L-P3 atom was easily deformed by the Si tip in Figure 3. If atom 1 was removed, the L-S4 atom could change to two-coordinated, which would make the L-S4 atom easy to deform. Because the L-P3 atom is located near the L-S4 atom and on the surface side compared to the L-S4 atom, the L-P3 atom can be deformed by the Si tip together with the L-S4 atom. In order to discuss whether atom 2 actually exists or not, we will consider the phase relationship of the rows of pentagon pairs on the upper and lower terraces, as shown in Figure 5 for two kinds of phase relationships (A and B). Here, phase A is the actual phase relationship of the 16×2 reconstruction, and phase B is the virtual one in which the row of pentagon pairs on the lower terrace are shifted by a half cycle (16×1). Here, we intentionally remove both atom 1 and 2, and as a result, the lower terrace becomes isolated from the upper terrace. In this case, we cannot distinguish phase A and B energetically because the positions of the second layer Si rows with respect to those of pentagons on the lower terrace are the same in either phase. Note that only phase A actually exists and the surface energy of phase A should be lower than that of phase B; that is, both atom 1 and 2 can not be removed simultaneously. Therefore, atom 2 must exist, however we could not observe it because the tip shape was simply insufficient. Figure 4b shows the atomic model which can explain the AFM images. It was found that atoms 1, 3, and 4 should be removed from the unreconstructed Si(110) surface containing atomic steps in order for the model and the AFM images to match. We could not find the accurate position of the pentagons, but it was confirmed that the pentagon consisted of five atoms, while in STM images, only four or five bright spots appear depending on the polarity of the tip bias.

Finally, we will compare our model (Figure 4b) with the universal building block (UBB) model of Si(110)-(16×2), which was recently proposed [38–39]. The feature of the UBB model at the step edge is that the atoms located at the edge of the first layer Si rows are the U-S1 and U-S5 atom (see Figure 4 in [39]), but the authors also suggest that there are still other possible configurations for the step edge. With respect to the step edge on the upper terrace, our model and the UBB model completely agree with each other. In particular, our experimental observation of the U-S4 and U-S5 atom directly prove it is valid that the U-S5 atom is located at the edge of the first layer Si row in the UBB model. In addition, the experimental observation of the L-S4 atom indirectly proves that it is valid for the U-S1 atom to be located at the edge of the first layer Si row in the UBB model because an additional atom next to the U-S1 atom would saturate the dangling bond of the L-S4 atom, which would contradict with the AFM image. With respect to the step edge on the lower terrace, the absence of atom 1 in our model was the discrepancy: in our model, atom 1 does not exist and atom 2 exists, while in the UBB model, both atom 1 and atom 2 exist. In our model, we removed atom 1 just to explain why the LP-3 atom was easy to be pulled up by the Si tip and we did not consider the energetic stability of the surface when atom 1 is removed. In the case of atom 2, we tried to prove the existence of atom 2 by an indirect explanation using Figure 5. To discuss whether atom 1 and atom 2 exist or not, and why phase B in Figure 5 has never been observed, further experimental and theoretical research will be needed.

## Conclusion

The Si(110)-(16×2) surface reconstruction was investigated by AFM at 78 K. We observed bright spots and pairs of pentagons on the upper and lower terraces, and directly confirmed that the pair of pentagons actually consisted of five atoms. A non-pentagonal structure in the 16×2 reconstruction and the disorder region were observed with high resolution, and the defect structure of the pentagon due to the lack of a P3 atom in the structure of pentagon was observed. We also observed the atoms of U-S4, U-S5 and L-S4 on the step edges which to date have not been observed in the previously proposed structural model. We proposed a structural model that explains the AFM images. Here it was concluded that atom 1, 3, and 4 should be removed from the unreconstructed Si(110) surface containing atomic steps. Among the atoms at the step edge, the U-S4, U-S5 and L-S4 atoms were not reported in previous STM studies. This work provides new evidence and a call for the further investigation of the surface reconstruction with atomic resolution on the Si(110) surface by AFM and opens up novel routes for studying its structure model.
